# Novel design of inspiratory flow generation and gas mixing for critical care ventilators suitable for rapid production and mass casualty incidents

**DOI:** 10.1038/s41598-023-34300-x

**Published:** 2023-05-02

**Authors:** Karel Roubik, Vaclav Ort, Lenka Horakova, Simon Walzel

**Affiliations:** Department of Biomedical Technology, Faculty of Biomedical Engineering, Czech Technical University in Prague, Prague, Czech Republic

**Keywords:** Biomedical engineering, Respiratory distress syndrome

## Abstract

Scarcity of medical resources inspired many teams worldwide to design ventilators utilizing different approaches during the recent COVID-19 pandemic. Although it can be relatively easy to design a simple ventilator in a laboratory, a large scale production of reliable emergency ventilators which meet international standards for critical care ventilators is challenging and time consuming. The aim of this study is to propose a novel and easily manufacturable principle of gas mixing and inspiratory flow generation for mechanical lung ventilators. Two fast ON/OFF valves, one for air and one for oxygen, are used to control the inspiratory flow generation using pulse width modulation. Short gas flow pulses are smoothed by low-pass acoustic filters and do not propagate further into the patient circuit. At the same time, the appropriate pulse width modulation of both ON/OFF valves controls the oxygen fraction in the generated gas mixture. Tests focused on the accuracy of the delivered oxygen fractions and tidal volumes have proved compliance with the international standards for critical care ventilators. The concept of a simple construction using two fast ON/OFF valves may be used for designing mechanical lung ventilators and thus suitable for their rapid production during pandemics.

## Introduction

Mass casualty incidents (MCI) have been defined by the Pan American Health Organization (PAHO) as “any event resulting in a number of victims large enough to disrupt the normal course of emergency and health care services”^1^. Natural disasters, man-made disasters such as war or bioterrorism and infectious respiratory diseases can cause situations where health care providers have limited resources available^2^. The recent COVID-19 pandemic can also be perceived as an MCI^3^ due to a global scarcity of critical care resources. In spring 2020, a shortage of ventilators was reported from Italy^4^ and this situation inspired many teams worldwide to attempt to design easily manufacturable ventilators utilizing several different approaches from readily available components. The most popular designs were based on a manual resuscitator^5^. This concept has already been used in the MIT emergency ventilator in which the mechanical paddles are driven by a small motor in order to push air into the lungs^6^. Other designs utilized pumps, screw compressors, pistons, etc^5^. An interesting approach to solving shortage of medical grade ventilators is repurposing existing CPAP machines by adding an intermediate module in the ventilation pathway between the CPAP machine and the patient. The module assures pressure cycling with a possibility to adjust several ventilatory parameters^7^.

However, even rapidly manufactured ventilators during pandemic situation must comply with the international standards for medical equipment, international standards for critical care ventilators^8^, and should allow setting of the ventilatory parameters according to the current lung protective ventilation guidelines^9–13^. When the simplified devices do not fulfill these criteria, they cannot be distributed to medical facilities and their use may be associated with severe adverse effects^14^.

The inspiratory flow generation in standard critical care ventilators can be based on several different mechanisms. The vast majority of mechanical ventilators comprises a proportional valve and a mixing chamber^15^. However, manufacturing of ventilators using this technical solution requires custom made pneumatic components, which were not available for rapid production of ventilators in order to defeat their shortage in hospitals during the recent COVID-19 pandemic situation. A design of a ventilator with proportional valves is usually time consuming and requires extensive testing just as turbine-based ventilators and ventilators that incorporates bellows or pistons. Even though the negative pressure generated by the turbine and bellows sucks the ambient air that can be contaminated by chemical substances or biological agents, these ventilators do not require high flow gas supply which can be appreciated during mass casualty incidents^16^, preventing an overload of gas distribution systems.

The aim of the study is to propose a novel and simple principle of gas mixing and inspiratory flow generation for rapid production of ventilators used during mass casualty incidents and to test whether the new concept can be utilized in ventilators compliant with the international standards mandatory for critical care ventilators.

## Methods

### Inspiratory flow generation

**Figure 1:**
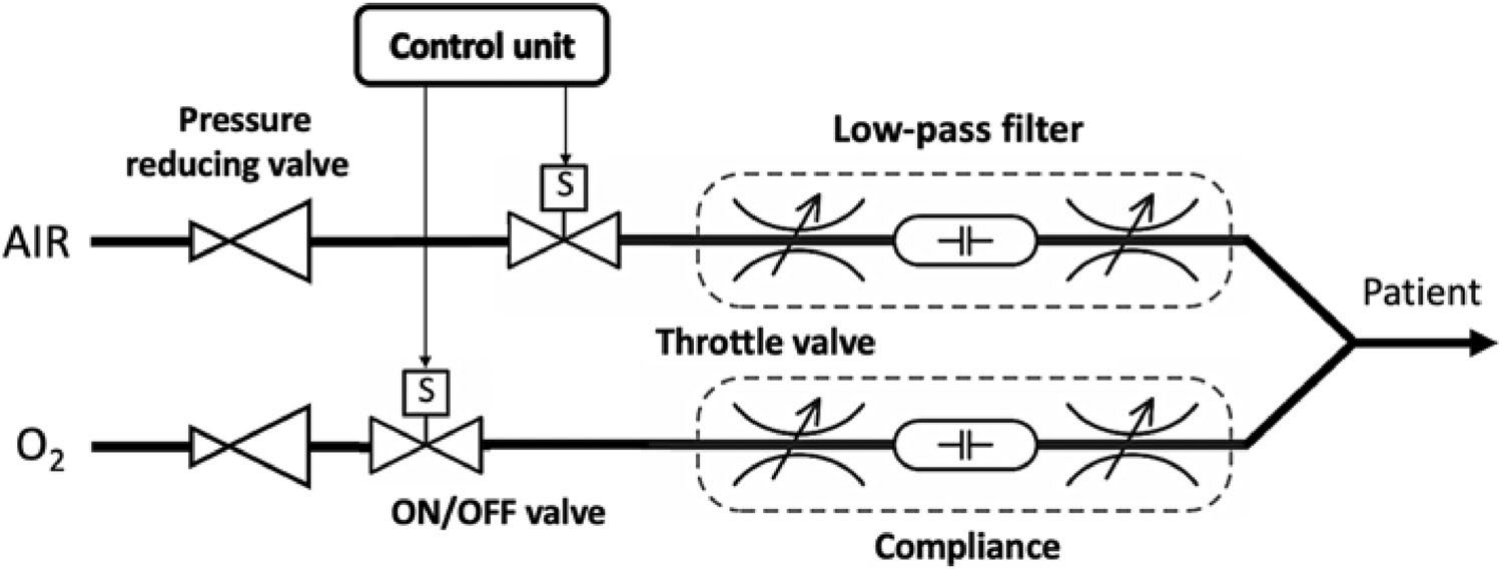
A scheme of inspiratory flow generation and gas mixing.

The generation of inspiratory flow is ensured by two independent branches, one for air and one for oxygen. Both inlet pressures are first reduced to the same working pressure by pressure reducing valves at the inlet of each of the branches, as shown in Fig. 1. The working pressures are then supplied to inputs of the fast, computer-controlled two-state ON/OFF valves. Acoustic low-pass filters are connected behind the ON/OFF valves to smooth out the short pulses generated by opening and closing the valves.

**Figure 2:**
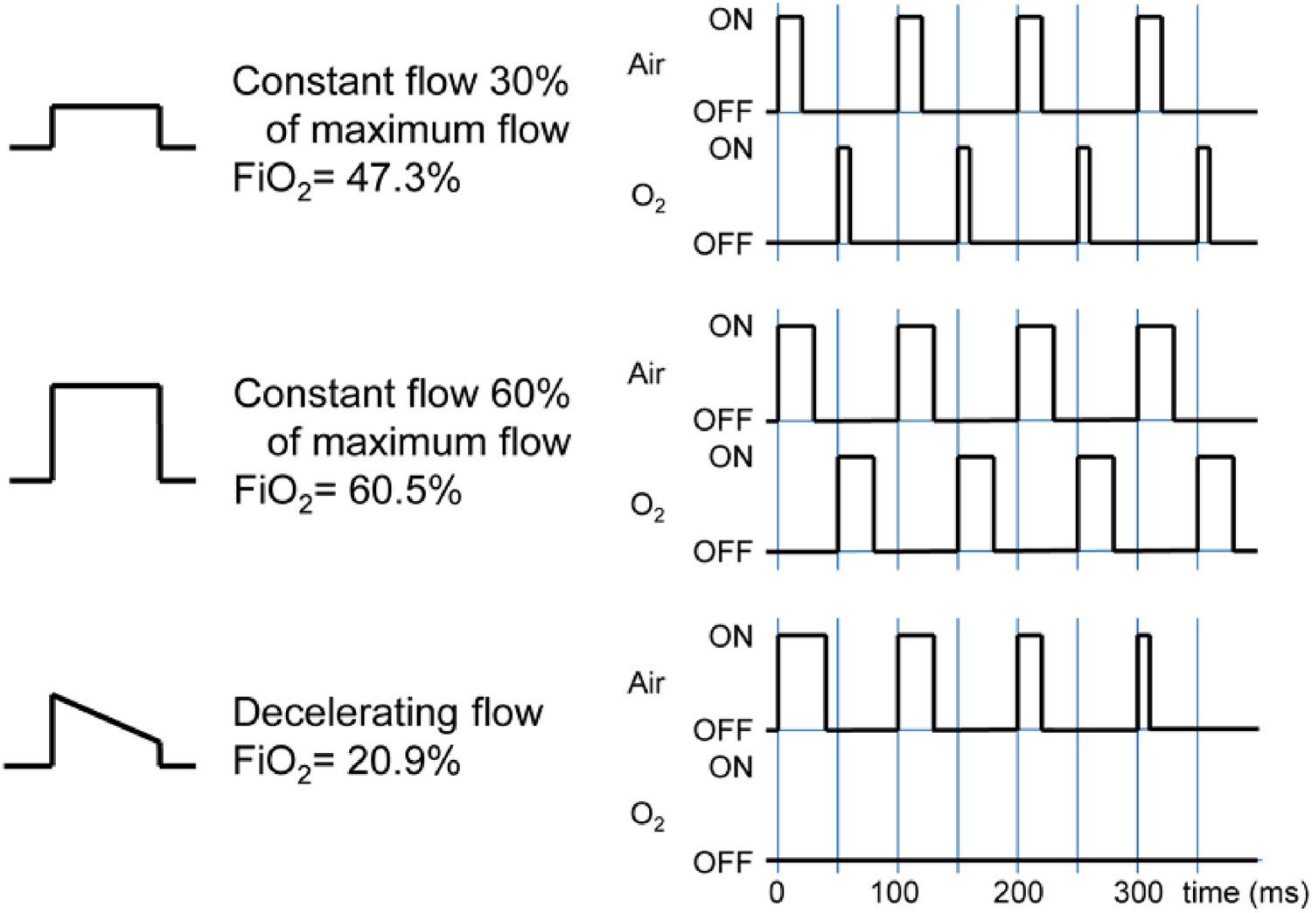
Examples of pulse sequences for generating inspiratory profiles with different flow patterns, magnitudes (i.e., tidal volumes) and fractions of oxygen in the gas mixture. Inspiratory profiles filtered by the low-pass acoustic filters are depicted on the left, the corresponding pulse sequences on the right.

The principle of pulse width modulation (PWM) is used to control the flow in each branch. Each of the ON/OFF valves switches between open and closed states with a working period of units or tens of milliseconds. Fast switching of the open and closed states of the ON/OFF valve causes a pulsatile gas flow behind the valve. The volume of the pulses is defined by the ratio of the open and closed state times in each branch. These pulses are then smoothed by a low-pass acoustic filter consisting of a combination of two flow resistances (represented by the throttle valves in Fig. 1) and a compliance connected behind the ON/OFF valve. By changing the ratios of the opening and closing times of the ON/OFF valve, it is possible to control the shape of the resulting filtered inspiratory flow and the total gas flow in a given branch, as depicted in Fig. 2. By combining the flows of both gases, it is possible to control the oxygen fraction in the gas mixture, the respiratory rate, the shape of the flow curve, and the overall tidal volume during inspiration.

For a smoother flow, the individual pulses of both ON/OFF valves are evenly distributed throughout the inspiration. The even distribution of pulses from both ON/OFF valves and the use of the low-pass acoustic filters in each branch result in a smooth flow of the ventilation mixture.

### Experimental ventilatory set

To test and evaluate the concept of gas mixing and inspiratory flow generation, an experimental ventilator was assembled. Air and oxygen inlet pressure reducing valves were set to the pressure of 200 ± 2 kPa. Both ON/OFF valves (MHJ9-QS-4-MF, Festo, Esslingen am Neckar, Germany) utilize PWM with constant switching frequency of 10 Hz.

The experimental ventilator is functionally based on a programmable logic controller CP6606 (Beckhoff, Verl, Germany) with ARM Cortex™-A8 processor. A main control algorithm of the experimental ventilatory set was developed in a programing environment TwinCAT (version 3.1, Beckhoff, Verl, Germany). The respiratory phases are hard controlled, i.e., all phases are strictly timed under the current setting which is controlled by an operator. Each change of the ventilation parameters setting causes a recalculation of the ON/OFF valves timing.

The low-pass acoustic filter following each ON/OFF valve is composed of a compliance represented by a rigid plastic tube with the inner diameter of 29 mm and length of 150 mm, i.e., its volume is approximately 100 mL, and two throttle valves (GRO-QS-6, Festo, Esslingen am Neckar, Germany) creating resistances. The first throttle valve in the direction of gas flow in each branch is set to regulate the flow of the respective gas to 55 L min^−1^ (± 0.5 L min^−1^) when the second throttle valve is fully opened. The second throttle valve is then adjusted to further limit the flow to 50 L min^−1^ (± 0.5 L min^−1^). The outputs from both low-pass acoustic filters are then connected using a T-connector. The resulting mixture flows to the inspiratory port using a tube with an inner diameter of 4 mm and a length of 600 mm, which further dampens remnants of oscillations in the pulsatile flow.

Cycling between inspiratory and expiratory phases was assured by a pneumatically driven expiratory valve. Expiratory valve closes the expiratory branch of the patient circuit during inspiratory phase and opens the branch during expiratory phase. During inspiratory phase, it works as a pneumatically controlled pressure relief valve as well, allowing to limit the maximum inspiratory pressure in the patient circuit (Plim). In the expiratory phase, it acts as a pneumatically controlled pressure relief valve. Therefore, the valve also controls the positive end-expiratory pressure (PEEP). The configuration of the whole system is presented in Supplementary Material 1.

### Testing

The new principle of gas mixing and inspiratory flow generation was subjected to a number of laboratory tests using an experimental ventilatory set which also comprised an airway pressure limit valve and an expiratory valve. The inlet pressures were set to 4.5 bar (both air and oxygen) as a standard value of pressures in hospital gas distribution systems according to the international standard for medical gas pipeline systems ISO 7396-1:2016^17^.

**Figure 3:**
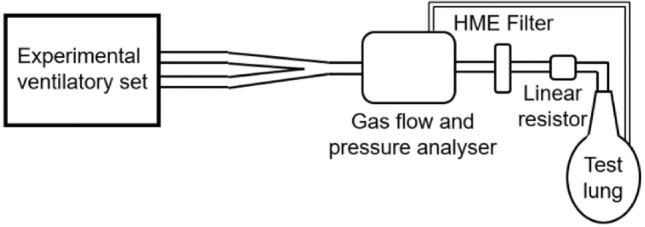
Measuring setup for assessment of the technical properties of the experimental ventilatory set.

The measuring setup presented in Fig. 3 was assembled according to the recommendation for testing of ventilators issued in the international standard for critical care ventilators^8^. Flow analyzer Fluke Biomedical VT900A (Fluke Biomedical LLC, WA, USA) was used to measure tidal volumes, pressures and oxygen concentrations. Heat and moisture exchanger (HME) filter (DAHLHAUSEN CZ, spol. s.r.o., Kurim, Czech Republic) was used as a standard component of the patient circuit. Linear resistor with a resistance of 5 cmH_2_O s L^−1^ (Model 7100R, Hans Rudolph, inc., Shawnee, KS, USA) simulated the resistance of the respiratory system.

As a test lung, a stable glass container with the nominal volume of 50 L (adiabatic compliance: 40.0 ± 0.04 mL cmH_2_O^−1^, isothermal compliance: 52.9 ± 0.15 mL cmH_2_O^−1^) was used. Measurement of compliances was conducted using a large volume calibration syringe (Hans Rudolph, inc., Shawnee, KS, USA) and a pressure measuring device (Fluke Biomedical VT900A, Fluke Biomedical LLC, WA, USA) according to study by Roubik et al.^18^.

The initial ventilatory parameters of the experimental ventilator wewe set as follows: positive end-expiratory pressure PEEP = 5 cmH_2_O, inspiratory to expiratory time ratio I:E = 1:2, pressure limit Plim was set to the maximum, i.e., 60 cmH_2_O.

The agreement between the measured FiO_2_ and the set FiO_2_, presented in percentages, was measured in the whole range of values between 21 and 100% in steps of 10%. Seven different tidal volumes were set for each FiO_2_ setting in the range of 225–675 mL (in steps of 75 mL) for RR = 20 breaths per minute and eight different tidal volumes were set for each FiO_2_ setting in the range of 225–400 mL (in steps of 25 mL) for RR = 35 breaths per minute. The measured FiO_2_ values are presented as mean ± standard deviation and compared with the preset FiO_2_.

The dependence of the delivered tidal volumes on the set FiO_2_ was tested at four tidal volume settings (225, 370, 525, 675 mL) at RR = 20 breaths par minute. Fraction of oxygen was set in steps of 10% in the range of 21–100%. Each measurement setting lasted 150 s (i.e., 50 breathing cycles). The measured delivered tidal volumes are presented as mean ± standard deviation.

In order to test the output characteristics of the experimental ventilatory set, the effect of resistance and compliance of the respiratory system model on the pressure and volume performance of the experimental ventilatory set was measured using a set of resistors and glass containers with different volumes. The glass containers with nominal volumes of 15, 25, 35 and 50 L (the adiabatic and isothermal compliances of these containers are presented in Table 1) were used, together with the added resistances of 5, 20 and 50 cmH_2_O s L^−1^. The FiO_2_ was set to 60% for all the settings. For every resistance and glass container combination, tidal volumes were set in the range of 225–675 mL in steps of 75 mL, if not limited by the airway pressure limit valve.

**Table 1:** Adiabatic and Isothermal compliance for each glass container used as a test lung.

Volume (L)	Adiabatic compliance (mL∙cmH_2_O^−1^)	Isothermal compliance (mL∙cmH_2_O^−1^)
15	11.9 ± 0.1	16.1 ± 0.1
25	19.3 ± 0.2	27.0 ± 0.1
35	25.4 ± 0.4	35.0 ± 0.3
50	40.0 ± 0.1	52.9 ± 0.2

The analysis of the dependance of the delivered tidal volume and maximum pressure on adiabatic compliance and resistance of the connected respiratory system model was performed using 3D plots. Each measurement lasted 60 s (i.e., 20 full breath cycles) and the average value for every configuration was calculated.

## Results

**Figure 4:**
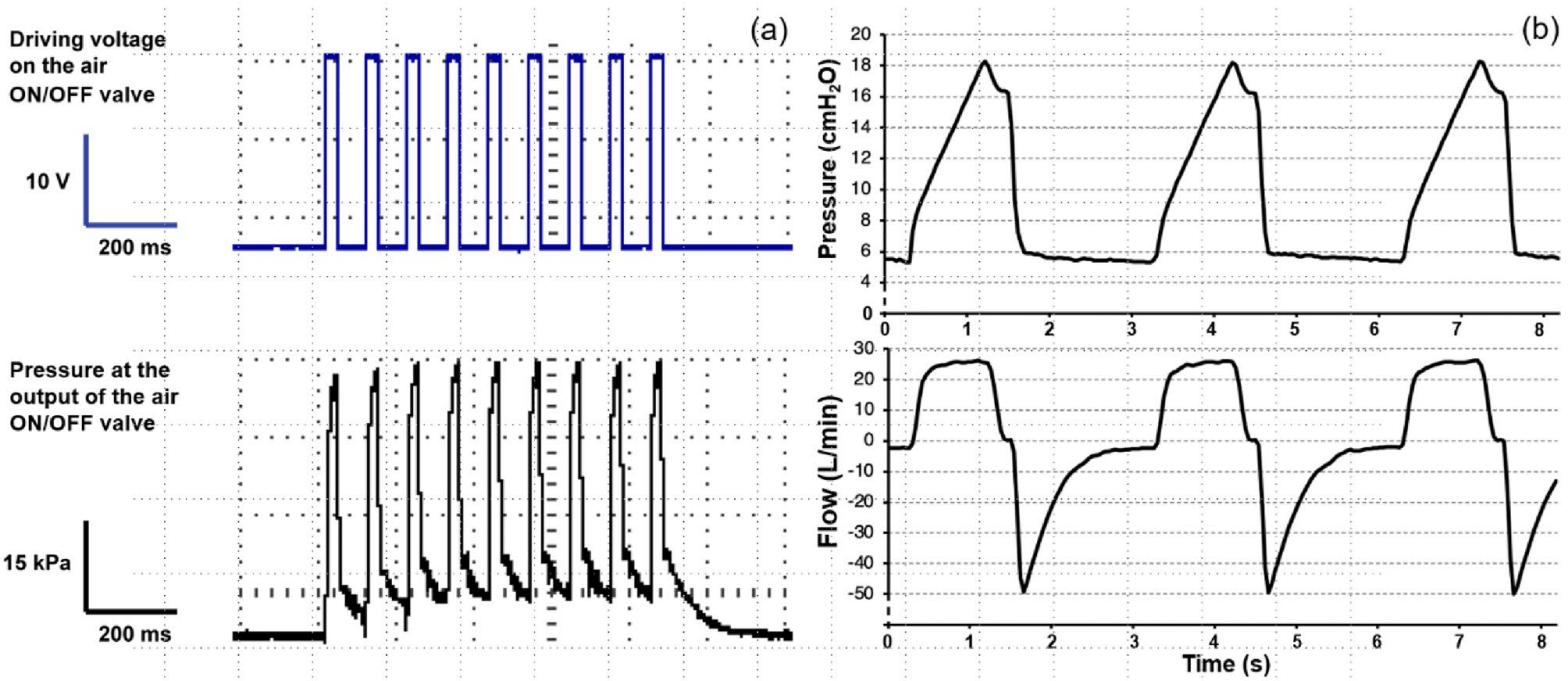
The recorded sequence of electric pulses and corresponding pressure pulses creating the inspiratory flow (**a**) and the resulting pressure and flow curves after the pneumatic filtering (**b**).

The fast opening and closing of both ON/OFF valves during inspiration cause generation of sequence of gas pulses as shown in Fig. 4a. The number and length of the pulses correspond to the set ventilatory parameters (respiratory rate RR = 20 min^−1^, inspiratory to expiratory time ratio I:E = 1:2, set tidal volume was 450 mL, fraction of oxygen in the inspiratory gas FiO_2_ = 60%). This fast sequence of short pulses of pressure are then smoothed by a low-pass acoustic filter, as shown in Fig. 4b, and mixed with the second branch. The introduced inspiration pause (15% of the total inspiratory time long) allows the plateau pressure to be measured. For this ventilation setting, the Pmax value was 18 cmH_2_O, the plateau pressure 16 cmH_2_O and the inspiratory flow was approximately 25 L min^−1^.

**Figure 5:**
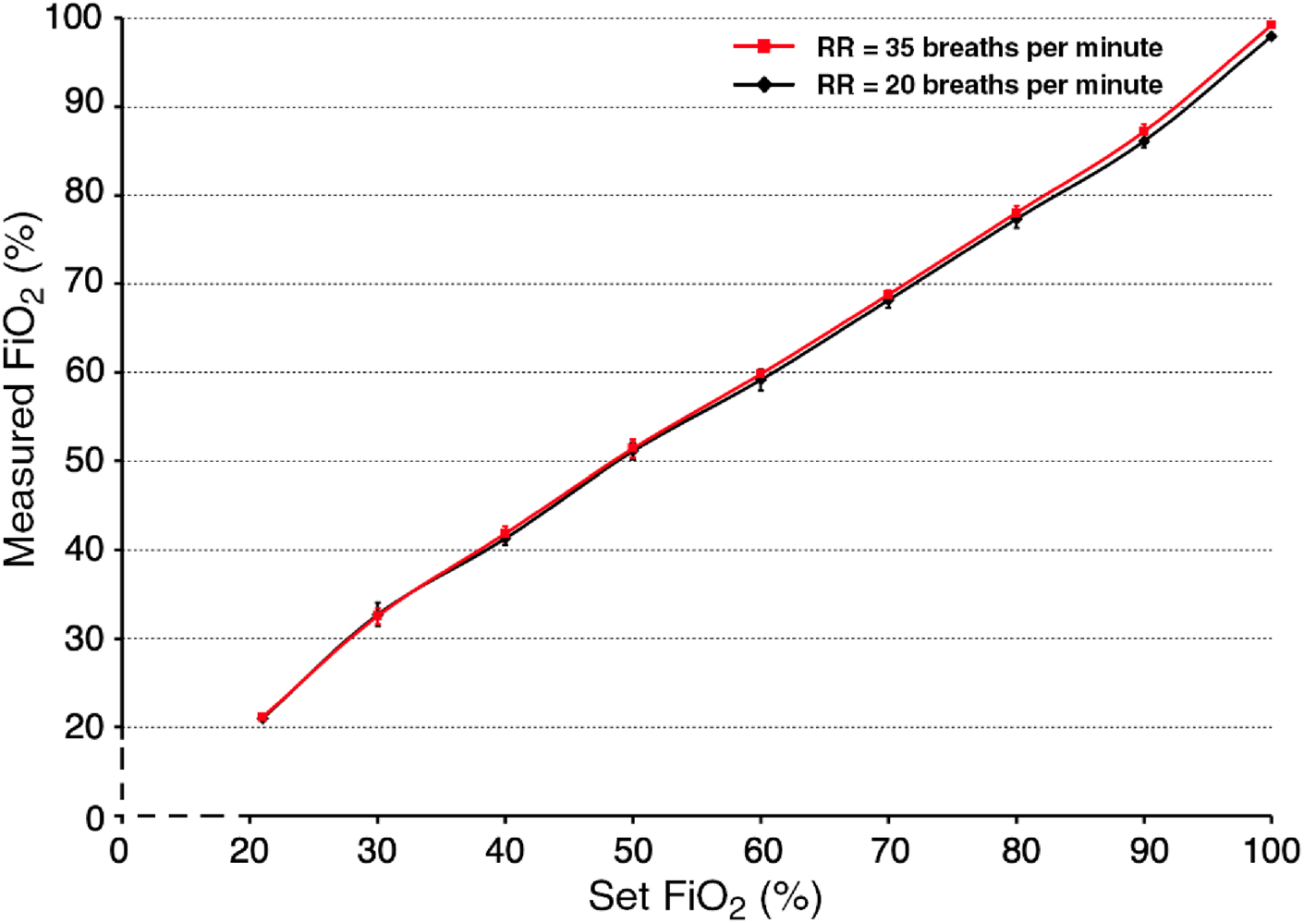
The dependance of measured FiO_2_ on the set FiO_2_ for RR = 20 breaths per minute (in black) and RR = 35 breaths per minute (in red).

The dependance of the measured FiO_2_ on the set FiO_2_ values is depicted in Fig. 5. The measured FiO_2_ value best corresponds to the preset FiO_2_ value at 60% with the average measured value of 59.2 ± 1.2% FiO_2_ at RR = 20 breaths per minute and 59.8 ± 0.5% FiO_2_ at RR = 35 breaths per minute. As the preset oxygen concentration in the inspired mixture decreases, a slightly higher oxygen concentration values are measured. Conversely, a slightly lower oxygen concentrations are measured as the oxygen concentration setting increases. For the preset 90% FiO_2_, 86.1 ± 1.3% FiO_2_ at RR = 20 breaths per minute and 87.2 ± 0.9% FiO_2_ at RR = 35 breaths per minute were measured. For the preset 30% FiO_2_, 32.7 ± 1.2% FiO_2_ at RR = 20 breaths per minute and 32.5 ± 0.9% FiO_2_ at RR = 35 breaths per minute were measured. At the 100% oxygen setting, FiO_2_ of 98% at RR = 20 breaths per minute and 99.2% at RR = 35 breaths per minute were measured for all tidal volumes. No effect of the measured oxygen concentration with increasing or decreasing tidal volume was found.

**Figure 6:**
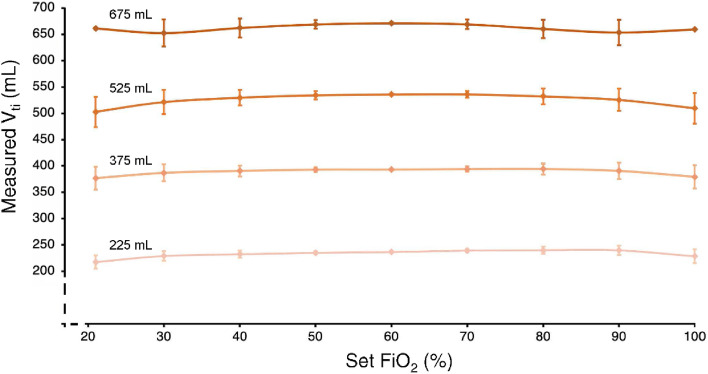
The dependance of the measured tidal volume V_ti_ on the set inspiratory oxygen fraction FiO_2_ and the set tidal volume.

The delivered tidal volume for nine different set FiO_2_ values at four different preset tidal volumes is depicted in Fig. 6. The ventilator delivers tidal volumes with the smallest standard deviation at FiO_2_ of 60%. Towards the extreme set values of the oxygen concentration, the average tidal volume slightly decreases, and the corresponding standard deviation increases. As the preset tidal volume increases, the standard deviation increases. The only exception occurs for the extreme values of the set FiO_2_ and the preset tidal volume of 675 mL, where the standard deviation is almost zero.

Additional tests were conducted in order to document stability of the generated inspiratory flow and its independence on increasing pressure in the expiratory limb of the patient circuit, and to test accuracy of delivered tidal volume at RR = 35 breaths per minute for three levels of FiO_2_ (30%, 60% and 90%). Results of the tests are presented in Supplementary Material 2.

**Figure 7:**
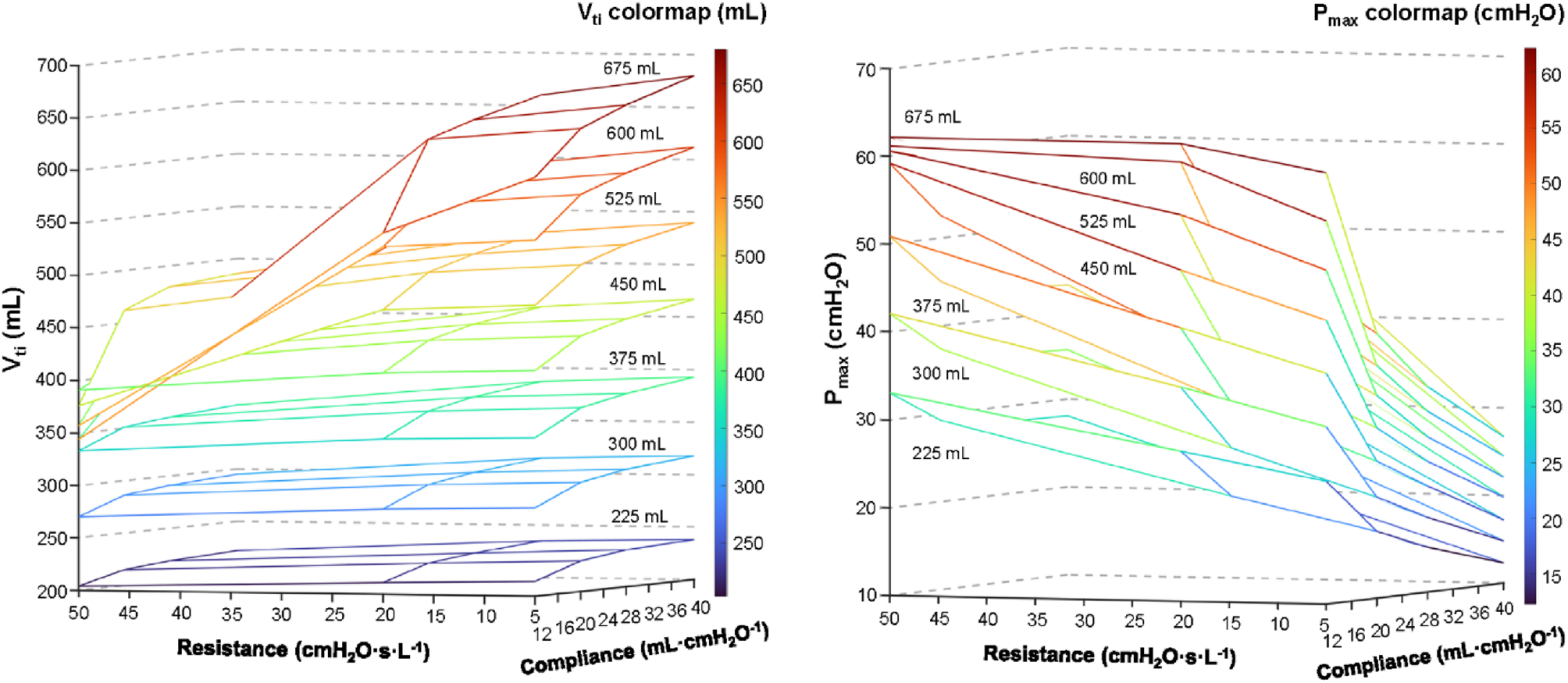
The 3D plots of the dependance of the delivered volume and maximum pressure on the compliance and resistance of the model of the respiratory system.

The effect of respiratory system resistance and compliance on the delivered tidal volumes and measured pressures is shown in Fig. 7. The largest delivered tidal volume for all the set tidal volumes was for the smallest resistance (5 cmH_2_O s L^−1^) and the highest compliance (40 mL cmH_2_O^−1^). The highest delivered tidal volumes were measured at the set tidal volume of 675 mL. The average delivered tidal volume was 680 mL at Pmax 27.5 cmH_2_O. Decreasing compliance and increasing resistance caused a decrease in delivered tidal volume and higher pressure for all the set tidal volumes. Higher set tidal volumes caused an increase in pressure till the limit of 60 cmH_2_O. During the pressure limitation, the tidal volumes decreased at the higher tidal volumes and thus the corresponding planes in the graph overlapped.

## Discussion

Results of the experimental measurements suggest that a pair of fast ON/OFF valves for air and oxygen, controlled by PWM, is suitable for generating inspiratory flow of various magnitude and flow profile and at the same time producing a desired air-oxygen mixture. Even though the inspiratory gas flow is generated as a sequence of short gas flow pulses, the pneumatic filtering suppresses the pressure swings effectively and they are not apparent on the flow leaving the ventilator into the patient circuit. The advantages of this solution are the simplicity of the design using ON/OFF valves that can only be in open and closed states, and the consequent simplicity of their control without the need to use feedback algorithms resulting in high reliability of the circuit. Another advantage is the significantly lower cost of ON/OFF valves compared to proportional valves, in the order of 5–10 times.

For practical realization of the inspiratory flow generator, such ON/OFF valves should be used which have a high switching frequency and for which the manufacturer guarantees a sufficient number of switching cycles, i.e., a long service life of the ON/OFF valves. According to the manufacturer of the ON/OFF valves we used (MHJ9-QS-4-MF, Festo, Esslingen am Neckar, Germany), the switching time to ON at 400 kPa input pressure and 24 V control voltage is 0.8 ms and switching time to OFF is 0.4 ms. For the input pressure of 50 kPa the switching time to ON is 0.7 ms and the switching time to OFF is 0.5 ms. In our case the manufacturer guarantees 5 billion switching cycles of the ON/OFF valves. Assuming the PWM control period of 100 ms and inspiratory to expiratory time ratio I:E = 1:2, the guaranteed number of cycles is sufficient for 47 years of continuous operation without any interruption. ON/OFF valves fast enough for this application and with a sufficient number of switching cycles were not available on the market a decade ago; nevertheless, there are currently several manufacturers offering suitable valves. The spectrum of usable valves is further limited because the ON/OFF valves certified to work with pure oxygen at high pressure must be used in the oxygen branch of the inspiratory flow generator. Other criteria must also be considered when selecting valves. These include biocompatibility requirements and the hazardous substances content in the materials used, which must be observed in view of the subsequent certification of the ventilator as a medical device. Other requirements should be met during construction of ventilator including the international standard for critical care ventilators (ISO 80601-2-12:2020) and related documents, especially in terms of safety features, monitoring, alarms etc.

International standard ISO 7396-1:2016 specifies the requirements for the design and installation of pipelines for medical gases, including oxygen and air, in healthcare facilities. According to this standard, the nominal supply pressure for oxygen and air should be 400–700 kPa (4–7 bar) at the point of use. Furthermore, the actual values of pressure may vary in time depending on the number of connected devices and their overall air or oxygen consumption. The pressure of 450 kPa used in our study is the typical pressure in medical air and oxygen distribution systems. As the inlet pressure can be in such a wide range and also as it can vary in time, the reduction of the inlet pressure to a stable working pressure is needed. The system assembled for the inspiratory flow generation in this study uses the working pressure of 200 kPa on the input ports of the ON/OFF valves. Considering the relatively high value of the working pressure together with the high flow resistance of the throttle valves connected, the configuration represents a constant flow generator with a high internal resistance. This was an intention to create a generator producing a constant flow that is minimally affected by the mechanical properties (resistance and compliance) of the connected respiratory system and which is also not affected by the inlet pressure variations. As a result, the flow rate generated (when the ON/OFF valve is open) is determined solely by the working pressure and the resistance of both throttle valves in series. As neither working pressure nor the throttle valve resistances are changing (they are adjusted during the manufacturing), the generated flow rate stays stable and equal to the factory-adjusted value. This flow rate is known to the ventilator software and therefore the software can modify the delivered tidal volumes simply by changing the time when the ON/OFF valves are open. Tidal volume generated is equal to the constant flow rate multiplied by the time when the valves are open. As inspiratory flow consists of several short pulses, the time for tidal volume calculation represents the overall time, i.e., the sum of all time periods when the ON/OFF valves are open during a single breath cycle.

In order to verify the concept and to describe the properties of the inspiratory flow generator, we conducted a performance test with different combinations of compliance and resistance levels of the respiratory system model, including extreme, clinically possible values. As the results in Fig. 7 show, the delivered tidal volume is only slightly dependent on the resistance and compliance. At the lowest set tidal volume of 225 mL with the smallest flow resistance (5 cmH_2_O s L^−1^) and the highest adiabatic compliance (40 mL cmH_2_O^−1^), the delivered tidal volume was 238 mL. The worst combination of respiratory model parameters (resistance of 50 cmH_2_O s L^−1^ and adiabatic compliance of 11.9 mL cmH_2_O^−1^) caused the decrease of the delivered tidal volume from 238 to 204 mL (14% decrease). The same relative decrease for the delivered tidal volume was identified for the set tidal volumes of 300, 375 and 450 mL where no pressure limitation occurred. The decrease in delivered tidal volume starts to be significant with a combination of an extremely high resistance (50 cmH_2_O s L^−1^) and a small adiabatic compliance (11.9 mL cmH_2_O^−1^). Nevertheless, the decrease in delivered tidal volume is caused by the activation of the airway pressure limit valve at 60 cmH_2_O (which is a safety requirement based on the international standard for critical care ventilators^8^) due to the excessively high pressure in the model of the respiratory system with an extremely unfavorable combination of mechanical parameters.

For the described method of inspiratory flow generation and mixing gases using two simple ON/OFF valves, the delivered tidal volume is well predictable. The ventilator software can determine the delivered tidal volume from the known value of the maximum flow rate (determined by the working pressure and resistance of the throttle valves) and the total time the ON/OFF valves are open during inspiration. Possible temporal instability of the working pressure or the presence of mechanical impurities in the pressure reducing valves or throttle valves can adversely affect the generated inspiratory flow and thus the delivered tidal volume. For this reason, it is advisable to include pressure and flow sensors in the patient circuit when using the described method of generating inspiratory flow^19^.

The tested principle of gas mixing of air and oxygen is sufficiently accurate. The measured FiO_2_ value best corresponds to the preset FiO_2_ value at 60%. This is caused by the identical 1:1 controlling PWM signal for both air and oxygen ON/OFF valves. Slightly higher oxygen concentration values (32.7%, 41.3% at RR = 20 breaths per minute and 32.5%, 41.9% at RR = 35 breaths per minute) were measured when lower oxygen concentrations were set (30%, 40%), and lower measured oxygen concentrations (77.3%, 86.1% at RR = 20 breaths per minute and 78.0%, 87.2% at RR = 35 breaths per minute) were measured when higher oxygen concentrations were set (80%, 90%). When setting the FiO_2_ extreme values (21% and 100%), the delivered concentrations are precise, because only one of the ON/OFF valves is activated. This performance is not a potential limitation of the proposed concept of gas mixing and inspiratory flow generation, but it is a result of the control algorithms and timing of the pulses. The computer used for the control of the valves is not able to change the length of the pulses for the ON/OFF valves continuously, but the minimum time span between the pulse length changes is 2 ms due to the actuation frequency of 500 Hz of the control algorithm. Inability to generate the pulse long precisely according to the theoretical calculation causes the observed imprecision of the FiO_2_ delivery. For certain FiO_2_ settings (FiO_2_ = 30% or 90%), in which shorter gas pulses are larger than expected, the resulting FiO_2_ is more affected. Despite the described deviations from the set FiO_2_ value, the measured FiO_2_ values are in accordance with the international standard for critical care ventilators^8^.

The experimental ventilator, incorporating the described principle, delivers the most stable tidal volumes with the smallest standard deviation at the set FiO_2_ of 60% when both the ON/OFF valves are controlled by the identical PWM signal. Absolute standard deviations are greater at higher volumes, but they are similar in relative numbers throughout the whole range of set tidal volumes. The explanation of this behavior is that the compliance in the acoustic filter is filled at a higher flow rate (55 L/min) than it is emptied (50 L/min). As a result, a certain volume of gas accumulates in it during the gas pulse delivery. This accumulated volume is gradually supplied to the inspiratory branch after the gas pulse has finished. This is the basic principle of gas pulse smoothing in the proposed acoustic filter. However, the volume of compliance is limited. For longer pulses, the compliance may become saturated and thus limiting the filling flow. As a result there is a slight reduction in the gas volume delivered by the pulse. At the set FiO_2_ of 60%, the accumulated gas volume is equal in both compliances resulting in the highest delivered tidal volume for the whole FiO_2_ setting range. At the set FiO_2_ of 21% and 100%, the accumulated gas is present only in one of the branches and that decreases the final delivered tidal volume. At the same time, with combinations of set ventilation parameters requiring an odd number of pulses within one breath, larger and smaller breaths alternate regularly. This is because the software alternates an even and odd number of pulses between the air and oxygen ON/OFF valve. This, in combination with the different PWM between the two ON/OFF valves, causes periodical alternation of two slightly different delivered tidal volumes. The exception is at the set tidal volume of 675 mL and FiO_2_ of 21% and 100% where combination of ventilation parameters requires an even number of pulses.

The mentioned behavior of the acoustic filters has a positive effect on proper mixing of air and oxygen and prevents the delivery of unmixed alternating pulses of air and oxygen to the patient's respiratory system. The compliances are capable of absorbing high-pressure short-time pulses of both gases. These compliances are emptied through the flow resistances determined mainly by the resistances of the second throttle valves and also by the flow resistance of the downstream connection to the patient circuit. The emptying of these compliances is slow and the flow rates of both gases leaving them to the subsequent parts of the ventilator are already fairly well smoothed. This means that even though the valves for air and oxygen are opened alternately and in pulses, at the outlet of the acoustic filter the flows are already smoothed and the gases are mixed simultaneously. For example, even though the air valve is open at any given time, oxygen is simultaneously being supplied to the patient circuit from the oxygen branch. Oxygen leaves the filter's compliance that was pressurized by the previous pulses of oxygen. In addition, at the end of the entire inspiratory branch, there is an inspiratory port of the ventilator, which is formed as a stainless steel cylindrical part with an internal volume of 20 mL. Two pipes with the administered air and oxygen enter this space through the side wall of the cylinder, thus providing additional mixing of the gases.

Despite the imprecisions in tidal volume generation described above, the experimental ventilator reliably delivers tidal volumes within the accuracy of ± ((15% from the set value) + 4 mL) according to the international standard for critical care ventilators^8^. All the described imprecisions in delivered tidal volumes and measured FiO_2_ are caused by the low constant switching frequency (10 Hz) of the ON/OFF valves. The only limitation for increasing the switching frequency was the used Beckhoff controller CP6606.

The selected working pressure and the throttle valves adjustment generating the maximum flow rate of 50 L min^−1^ and the cycling frequency 10 Hz were sufficient to cover the required range of delivered tidal volumes when constant inspiratory flow was used. If there is a demand for higher inspiratory flow rate (e.g., for generating a decelerating flow pattern), the maximum flow rate can be increased simply either by increasing the working pressure or by reducing the resistance of the throttle valves. From the engineering point of view, increasing the working pressure is a more preferable way as it further advances properties of the inspiratory flow generator by making it even less dependent on the connected respiratory system parameters (airway resistance and compliance). Changing resistances of the throttle valves would affect the time characteristics of the acoustic filters and therefore their tuning would be required. By setting the maximum flow rate of both gases, it is possible to adapt the desired range of tidal volumes to pediatric patients or to large animals as well.

Experimental verification of the inspiratory flow generator was performed only during volume-controlled ventilation, whose technical implementation is very simple, easy to control and easy to program. The authors speculate that if the flow generation method described above were used with a feedback control, it would be possible to create a source of constant inspiratory pressure, which is required to implement pressure-controlled ventilation. A feedback loop controller would have to react in real time to instantaneous changes in pressure during inspiration and adjust the generated flow rate to keep the pressure in the respiratory system constant. This is a common way to solve this task, however, the authors have not tested this approach.

The described design of inspiratory flow generation was successfully incorporated into a ventilator which was manufactured in the Czech Republic under a brand name CoroVent. This ventilator was approved for an emergency use in the Czech Republic and also acquired the Emergency Use Authorization (EUA) by FDA^20^, requiring a wide range of adjustable ventilatory parameters. CoroVent was distributed to Czech hospitals. The development of the COVID-19 pandemic revealed that the number of skilled caregivers and intensive care unit spaces rather than available ventilators posed the patient care limitation in many countries^21^. However, ventilator CoroVent was used for ventilation of COVID-19 patients in several exceptional cases in the event of ventilator shortage in the Czech Repubic.

## Conclusions

A couple of fast ON/OFF valves, one for oxygen and one for air, may be used for designing of easily manufacturable mechanical lung ventilators. The shape of the generated inspiratory gas profile and thus delivered tidal volume may be modified by the pulse width modulation sequence. When the control pulses are reasonably distributed between the air and oxygen valves, the desired fraction of oxygen in the inspiratory gas mixture can be reached. The principal advantage of this concept is its simple construction not requiring custom made components and time demanding development which suggests it for a rapid production, for example during pandemics.

## Supplementary Information

Supplementary Information 1.

Supplementary Information 2.

## Data Availability

The datasets generated and analyzed during the current study are available in the repository at https://ventilation.fbmi.cvut.cz/data/.
