# Supplementary material for: Novel design of inspiratory flow generation and gas mixing for critical care ventilators suitable for rapid production and mass casualty incidents
Source: Sci Rep. 2023 May 2;13:7153. doi: 10.1038/s41598-023-34300-x (PMC10153782; doi:10.1038/s41598-023-34300-x)
Supplement: Supplementary file 1 — Supplementary Information 1. [file 41598_2023_34300_MOESM1_ESM.pdf]

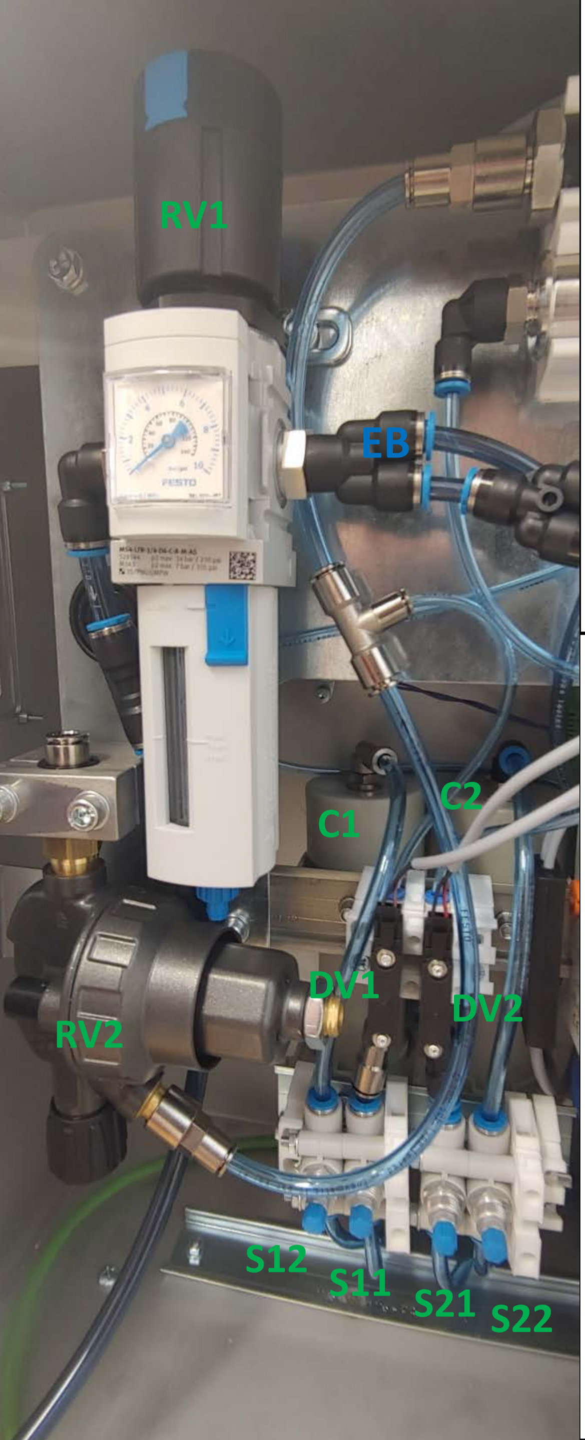

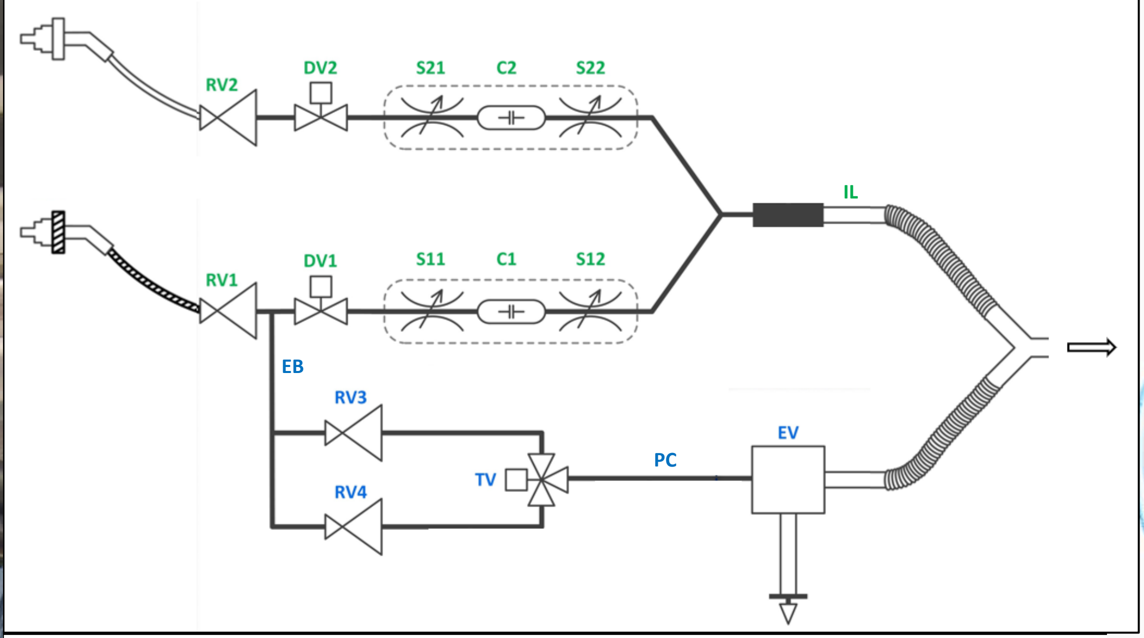

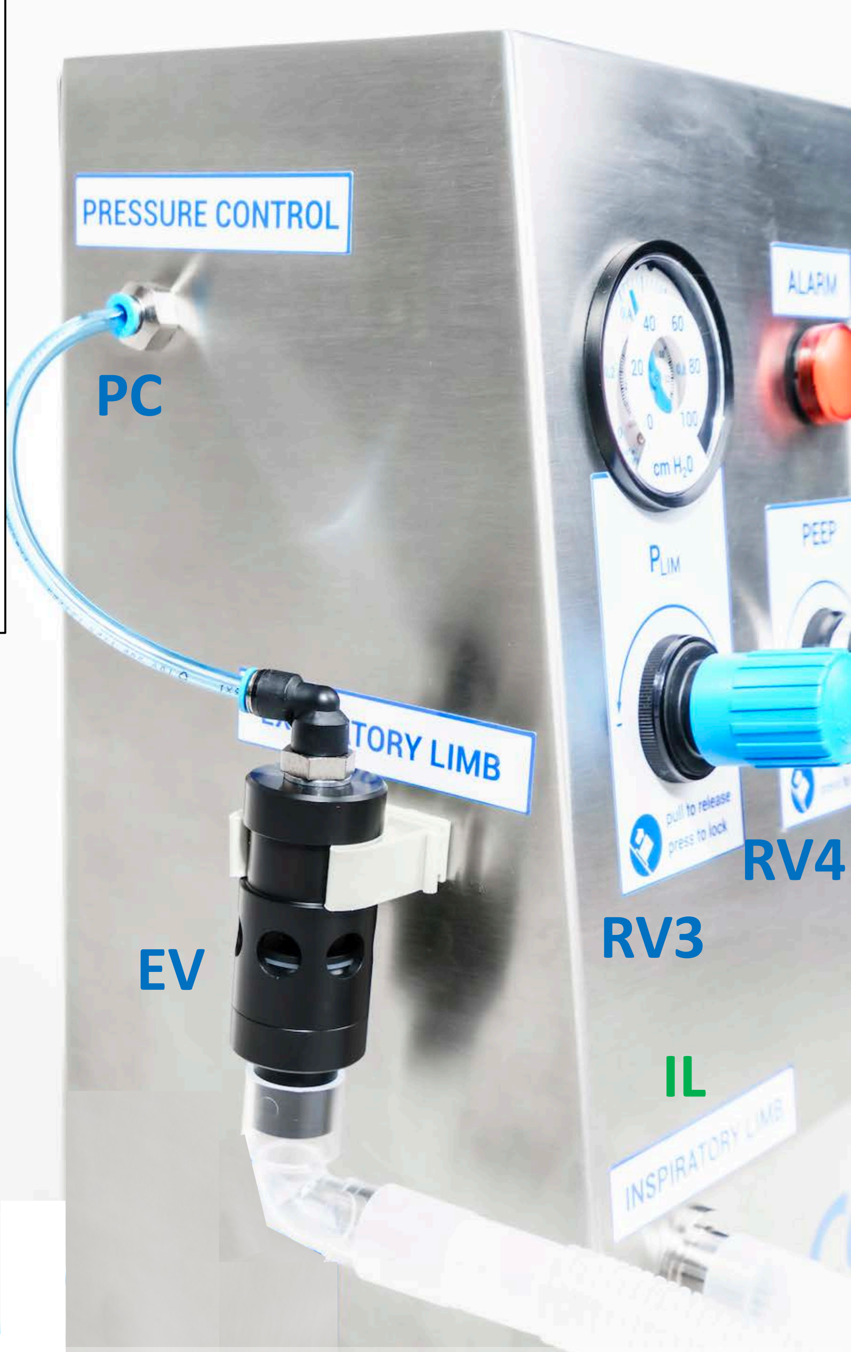

RV1	–	Air pressure reducing valve
RV2	–	Oxygen pressure reducing valve
DV1	–	ON/OFF valve in air branch
DV2	–	ON/OFF valve in oxygen branch
S11	–	First throttle valve in oxygen branch
C1	–	Compliance in oxygen branch
S12	–	Second throttle valve in oxygen branch
S21	–	First throttle valve in air branch
C2	–	Compliance in air branch
S22	–	Second throttle valve in air branch
IL	–	Inspiratory limb
RV3	–	Pressure reducing valve for Plim setting
RV4	–	Pressure reducing valve for PEEP setting
TV	–	3/2 valve
EV	–	Expiratory valve
PC	–	Pressure control
EB	–	Expiratory branch

Supplementary Material 1: Roubik, K. et al.: Novel design of inspiratory flow generation and gas mixing for critical care ventilators suitable for rapid production and mass casualty incidents. Scientific Reports, 2023.
