# Supplementary material for: Novel design of inspiratory flow generation and gas mixing for critical care ventilators suitable for rapid production and mass casualty incidents
Source: Sci Rep. 2023 May 2;13:7153. doi: 10.1038/s41598-023-34300-x (PMC10153782; doi:10.1038/s41598-023-34300-x)
Supplement: Supplementary file 2 — Supplementary Information 2. [file 41598_2023_34300_MOESM2_ESM.pdf]

Supplementary Material 2

Novel design of inspiratory flow generation and gas mixing for critical care ventilators suitable for rapid production and mass casualty incidents

Karel Roubik[1*], Vaclav Ort[1], Lenka Horakova[1], Simon Walzel[1]

*This **Supplementary Material 2** contains results of additional experiments characterizing performance of the proposed ventilatory system:*

1. Effect of increasing PEEP on inspiratory flow generation

To document stability of the generated inspiratory flow and its independence on increasing pressure in the expiratory limb of the patient circuit, we have performed experiments in which we evaluated the effect of increasing PEEP on the inspiratory pressure waveform and delivered tidal volume. We used the same set-up as described in the manuscript, but PEEP was increased gradually from 0 cmH_2O to 20 cmH_2O in steps of 5 cmH_2O. The ventilator setting was RR = 20 breaths per minute, I:E ratio 1:2, set tidal volume V_{ti} = 425 mL. Each pressure waveform presented is an average of four consecutive breath cycles.

The results are presented in SFig. 1 in the original pressure scale (upper graph) and also normalized (the bottom graph) to emphasize the possible changes in the generated waveform. We did not detected any significant effect of increasing PEEP on the inspiratory pressure pattern.

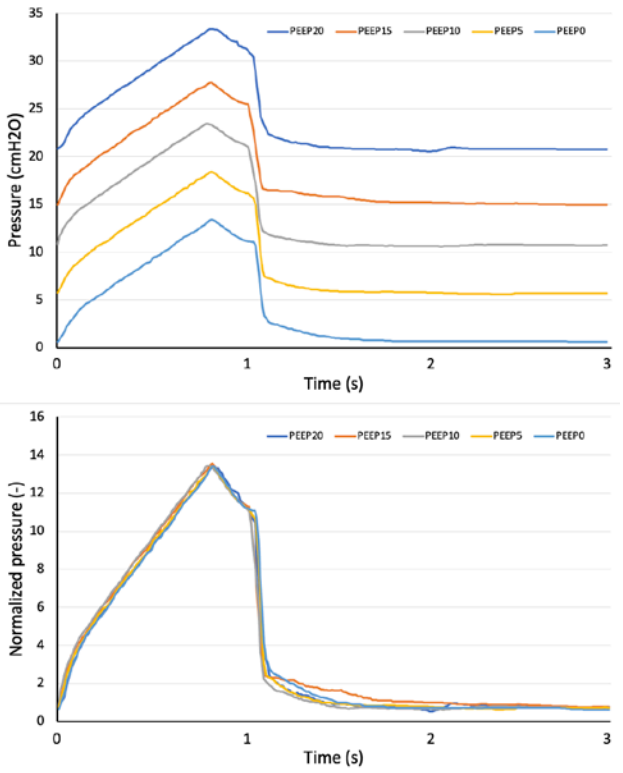

SFig. 1: Pressure waveforms at different PEEP levels in original pressure scale (upper graph) and normalized (the bottom graph).

Next, delivered tidal volume was measured during increasing PEEP from 0 to 20 cmH_2O. Presented tidal volumes are average values of four consecutive breath cycles. According to STab. 1, PEEP level did not affect the delivered tidal volume.

STab. 1: Dependence of generated inspiratory and expiratory tidal volumes on PEEP level.

PEEP	V_{ti} (mL)	V_{te} (mL)
0	431 ± 1	425 ± 2
5	430 ± 1	427 ± 16
10	430 ± 1	423 ± 9
15	429 ± 1	427 ± 9
20	429 ± 1	419 ± 14

2. The accuracy of delivered tidal volume V_{ti} on set FiO_2 (30%, 60% and 90%) at RR = 35 breaths per minute

In order to test accuracy of delivered tidal volume at RR = 35 breaths per minute, the similar setup was used as in the main manuscript. Three levels of FiO_2 were used: 30%, 60% and 90% at RR = 35 breaths per minute and I:E time ratio 1:2.

The experimental ventilator reliably delivered tidal volumes within the accuracy of ± ((15% from the set value) + 4 mL) required by the international standard for critical care ventilators (ISO 80601-2-12:2020) as documented in SFig. 2.

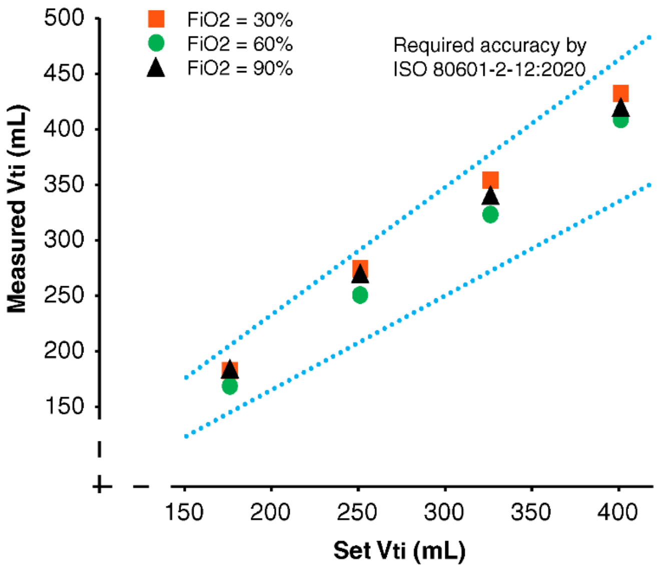

SFig. 2: Measured V_{ti} versus set V_{ti} for three values of FiO_2 at RR = 35 breaths per minute and I:E time ratio 1:2. The blue dotted lines represent limits of accuracy for delivered tidal volumes required by international standard ISO 80601-2-12:2020 for critical care ventilators.
